# A Simple Suturing Technique for Laparoscopic Ligation of Vascular Pedicles

**DOI:** 10.1155/DTE.2.121

**Published:** 1996

**Authors:** Keith A. Aqua, Joel M. Childers

**Affiliations:** University of Arizona Department of Obstetrics and Gynecology Division of Gynecologic Oncology Tucson Arizona 85712 USA

## Abstract

We report on the performance of 348 adnexectomies and 35 uterine artery ligations for both benign and malignant disease using a simple laparoscopic suturing technique. Only 5-mm ports are required, and there was no morbidity directly associated with this approach. The procedure can be performed quickly, is relatively inexpensive, and allows hysterectomy and oophorectomy to be performed without bipolar electrocautery.


*Diagnostic and Therapeutic Endoscopy*, 1996, Vol. 2, pp. 121–127
Reprints available directly from the publisher
Photocopying permitted by license only

© 1996 OPA (Overseas Publishers Association)
Amsterdam B.V. Published in The Netherlands
by Harwood Academic Publishers GmbH
Printed in Singapore

### A Simple Suturing Technique for Laparoscopic Ligation of Vascular Pedicles

KEITH A. AQUA and JOEL M. CHILDERS

*University of Arizona, Department of Obstetrics and Gynecology Division of Gynecologic Oncology, Tucson, Arizona*

*(Received June 14, 1995; in final form July 3, 1995)*

We report on the performance of 348 adnexectomies and 35 uterine artery ligations for both benign and malignant disease using a simple laparoscopic suturing technique. Only 5-mm ports are required, and there was no morbidity directly associated with this approach. The procedure can be performed quickly, is relatively inexpensive, and allows hysterectomy and oophorectomy to be performed without bipolar electrocautery.

**KEY WORDS:** Hysterectomy, oophorectomy, suturing techniques

### INTRODUCTION

Modern equipment and instrumentation have expanded the role of laparoscopy and have made this minimally invasive alternative to laparotomy an attractive operative tool for gynecologists. Whether alone or together, hysterectomy and removal of the adnexa are two of today's most commonly performed gynecological procedures. Ligation of vascular pedicles is required for these procedures. Endoscopic ligation of vascular pedicles has been accomplished with linear stapling devices, with desiccation using bipolar electricity, with multifire disposable clip applicators, and even with the carbon dioxide laser.[1–6]

In general, endoscopic suturing of vascular pedicles is more difficult to master and requires more time than the previously mentioned techniques. Nonetheless, we believe that the acquisition of endoscopic suturing skills allows the surgeon to be more versatile and potentially more cost-effective. We report on the use of an endoscopic suturing technique that can be performed quickly and safely and can be utilized on both the ovarian and uterine vessels. This "grasper-through-the-loop" technique requires no needles or intracorporeal knot-tying and provides uniformly good results for patients.

Address for correspondence: Joel M. Childers, M.D., 2625 North Craycroft Rd., Ste. 201, Tucson, AZ 85712.

### MATERIALS AND METHODS

### Subjects

A retrospective chart review was carried out for patients who underwent laparoscopic adnexectomy or hysterectomy or both between November 1990 and November 1994. Only patients who had the uterine artery or the infundibulopelvic ligament, or both, ligated using our endoscopic suturing technique were considered eligible for inclusion. The study group is divided into two groups: those in whom the ovarian vessels were sutured and those in whom the uterine vessels were sutured. Some subjects had both of these vascular pedicles sutured and therefore were included in both groups. Overall, the ages of the patients ranged from 29 to 93 years, with a mean of 59.4. Their weights ranged from 92 to 338 pounds, with a mean of 151.4.

The ovarian vessel ligation group was the largest, consisting of 200 patients. The ovaries were removed bilaterally in 148 (74%) of these patients and unilaterally in 52 (26%). Overall, there were 348 adnexa removed. The adnexa were extirpated in patients with adnexal masses,[64] during surgical staging for endometrial,[65] cervical,[11] and ovarian[19] carcinomas, and incidentally.[41] The uterine vessel ligation group included 38 subjects who underwent laparoscopically either total or supracervical hysterectomy. These hysterectomies were performed for endometrial carcinoma,[14] ovarian carcinoma,[2] in conjunction with adnexal masses,[10] and other benign diseases.[9]

Preoperatively, all patients gave complete history and underwent physical examinations. Routine preoperative laboratory evaluations also were performed. Antimicrobial prophylaxis was administered preoperatively to all patients in whom total hysterectomy or colpotomy incision was anticipated. The procedures were performed at one of three local hospitals.

### Procedure

A four-trocar technique is utilized. The primary port (10-mm) is placed in the umbilicus; 5-mm ancillary ports are placed bilaterally, under direct visualization, in the lower abdomen, lateral to the rectus abdominis muscles and the inferior epigastric vessels. The fourth ancillary port (5 to 12 mm) is placed in the midline, approximately 1 to 3 cm above the pubic symphysis.

The telescope is placed in the umbilical port and is held by the assistant, who uses a grasper placed through the lateral port on the side on which the adnexectomy is to be performed. The surgeon uses a grasper through the lower midline port and scissors with monopolar cautery capability through the contralateral lateral port. Adnexectomy is performed in the same manner on both sides of the pelvis.

### Removal of Left Adnexa

We will describe removal of the left adnexa. The surgeon stands on the patient's right side. The peritoneum over the psoas muscle is grasped and incised. The incision is extended parallel to the infundibulopelvic ligament. Monopolar cautery using the scissors as the source of electricity is used for hemostasis if necessary. The medial or posterior leaf of the broad ligament is retracted medially, opening the broad ligament. The ureter is visualized either directly in the open retroperitoneal space or indirectly through the medial leaf of the broad ligament. A window is created in the broad ligament below the ovarian vessel and above the ureter by grasping the peritoneum and cutting it with scissors. This window is then enlarged bluntly (Fig. 1).

The surgeon then manages the camera with his left hand and retracts the broad ligament with the suprapubic grasper. The assistant places a 30- to 36-inch 0 or 2-0 silk suture through the left lateral port, through the window in the broad ligament, and into the cul-de-sac. The grasper is removed from the window, reaches around the infundibulopelvic ligament, and into the cul-de-sac to grasp the suture (Fig. 2). The suture is removed through the ipsilateral port. Extracorporeal knots are tied using the Clarke knot-pusher (Fig. 3). The suture is cut and removed from the port. A prefabricated slipknot is placed through the same left lateral port. The grasper from the suprapubic port is placed through the loop of the pretied slipknot and grasps the pedicle distal to the previously placed silk suture. The surgeon then transects the ovarian vessel between the grasper and the suture ligature (Fig. 4). The assistant then brings the loop down and around the grasping forceps to ligate the pedicle. If the adnexa are not large, this loop may be placed around the utero-ovarian pedicle. This will save a step and a loop suture if adnexectomy is performed without hysterectomy. The suture is cut, allowing the assistant to remove the suture and insert another prefabricated slipknot. The surgeon passes the grasping forceps through the loop and grabs the proximal stump of the infundibulopelvic ligament. The slipknot is drawn superiorly around the tissue, allowing this pedicle to be doubly ligated (Fig. 5). The suture is cut, allowing the assistant to place the graspers through the port. If just the adnexa are to be removed, the utero-ovarian ligament is cut and the second slipknot placed on this pedicle. The specimen can now be removed. At the end of the procedure, the operative site is irrigated and inspected carefully to ensure adequate hemostasis.

In contradistinction to the ovarian vessel, the uterine vessels are suture-ligated through the suprapubic port. After the uterine arteries have been well skeletonized bilaterally and the bladder has been adequately mobilized off the pubovesical cervical fascia, a prefabricated slipknot is placed through the suprapubic port. A grasper placed through the ipsilateral abdominal port is placed through the loop and over the uterine vessels, *i.e.*, right port for right-side vessels. As performed at laparotomy, the grasper is placed perpendicular to the cervix. The grasper is closed, allowing the tip to slide off the cervix, clamping the uterine vessels (Fig. 6). This allows the pedicle to be taken adjacent to the cervix. Displacement of the corpus toward the contralateral side aids in visualization and is easily accomplished with a uterine manipulator. The uterine vessels are then transected with scissors placed through the contralateral port (Fig. 7). The loop is then slipped around the grasper and the uterine vessels are securely ligated. This is accomplished without back-clamping the uterine vessels, but is always performed after transection of either the infundibulopelvic ligament or the utero-ovarian ligament bilaterally, and after the contralateral uterine vessels have been skeletonized and are ready to be suture-ligated.

### RESULTS

Successful ligation and transection of the ovarian and uterine vessels were accomplished laparoscopically in all patients. Estimated blood loss during ligation of the infundibulopelvic ligament was unmeasurable except in a few patients, in whom the ovarian pedicle slipped out of the grasper. Even in these instances, the estimated blood

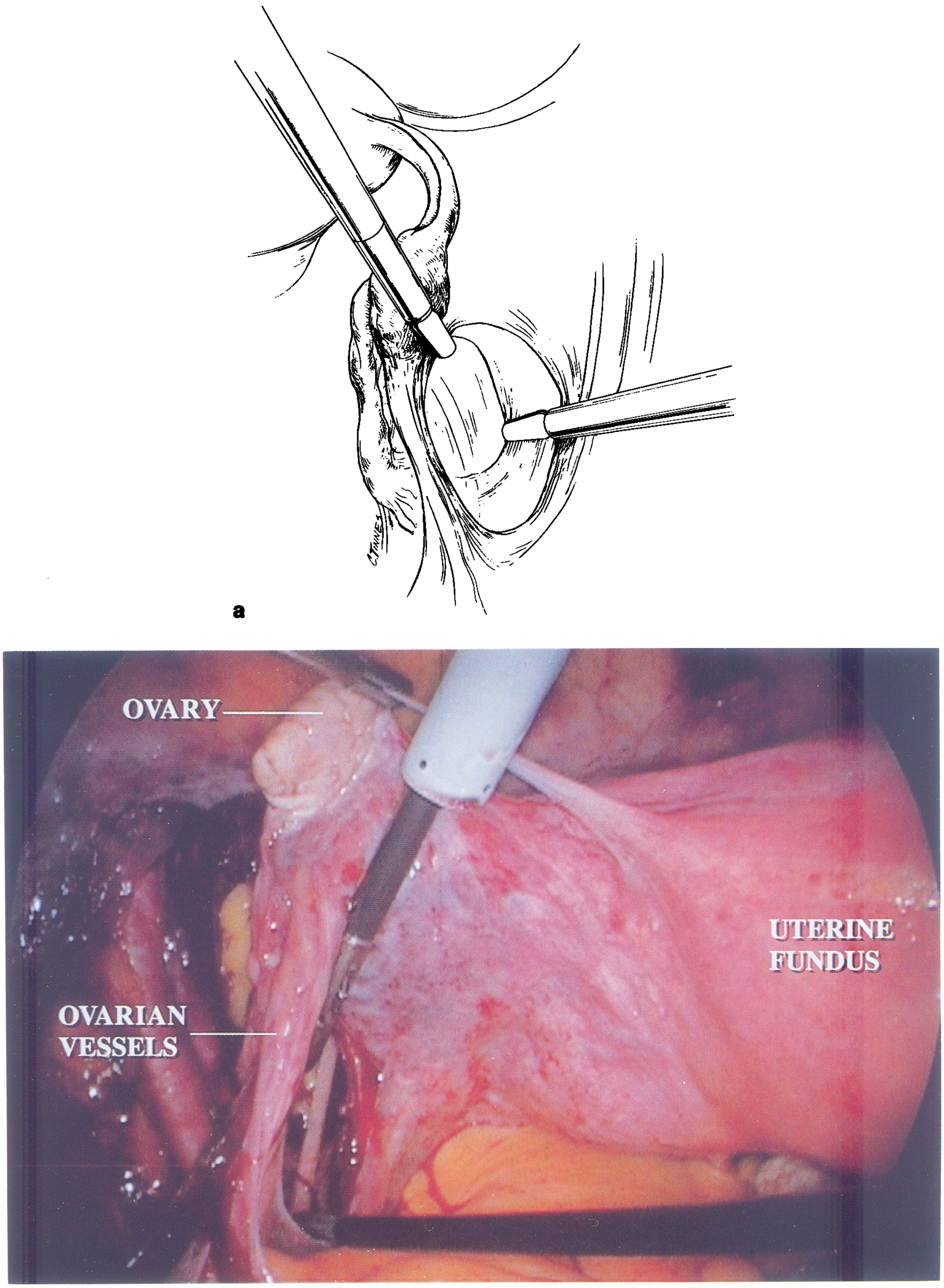

**Figure 1** A. A window in the anterior and posterior leaf of the right broad ligament has been created by sharp and blunt dissection. This window is below the infundibulopelvic ligament and above the right ureter. It is important to make this window as large as possible to facilitate the remaining steps in the procedure. B. This photograph demonstrates the creation of a window in the left broad ligament. The assistant elevates the ovary. The surgeon, standing on the right side, uses graspers placed through the suprapubic port (top) and scissors placed through the right lateral port (bottom).

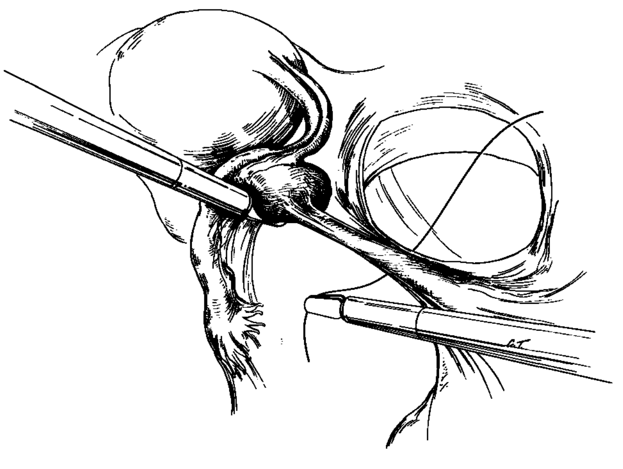

**Figure 2** A long silk tie is placed through the window and around the infundibulopelvic ligament. The tie is inserted through the ipsilateral lateral port.

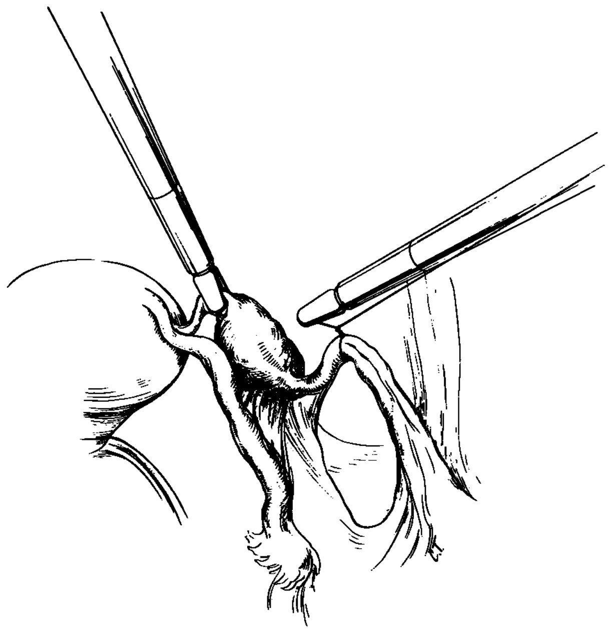

**Figure 3** Using extracorporeal knot-tying techniques, the infundibulopelvic ligament is suture ligated.

loss was <100 ml. Estimated blood loss during ligation of the uterine arteries ranged from 0 to 350 ml, with a mean <50 ml. The source of bleeding in those instances in which it was >100 ml was not back bleeding, but instead came from cutting too far beyond the tip of the grasper. In these circumstances, a second pedicle, usually the cardinal ligament, was grasped, transected, and ligated using the same technique. No blood transfusions were required in any patient. There were no injuries to bowel, bladder, or ureter with this method. There were no instances of infection of the sutured pedicles. No patient was taken back to the operating room for bleeding of the infundibulopelvic, utero-ovarian, or uterine pedicles. The last 25 infundibulopelvic ligaments ligated and transected in this series were timed from beginning of the peritoneal incision to completion and transection of the infundibulopelvic ligament. Times ranged from 3 to 12 minutes, with a mean of 5.4 minutes/side.

### DISCUSSION

Procedures that were once considered possible only via laparotomy are now commonly accomplished laparoscopically. Gynecologists currently perform transection of vascular pedicles by a variety of methods, including suture ligatures, stapling devices, and electrocautery. Each method has its advocates as well as its advantages and disadvantages. Stapling devices, although rapid, require large ports. The incidence of incisional bowel herniation through laparoscopic ports increases with increasing port size.[7–9] In addition, the large, nonarticulating stapling devices cannot always be properly placed around vascular pedicles. Ureteral injury has been reported during transection of the uterine artery using the stapling devices.[11,12] These disposable stapling devices are also expensive.

Bipolar cautery can be used through small ports and is inexpensive. Its major disadvantages include the risk of thermal damage to adjacent structures as well as reliance on properly functioning electrical surgical units, electrical cable, and the bipolar unit itself. The electrodes (paddles) not infrequently adhere to tissue. In addition, the smoke produced can obscure visualization and potentially increase blood levels of methemoglobin.[12]

The technique we describe is safe, easy, quick, and relatively inexpensive. Two prefabricated slipknot suture applicators are required for each ovarian pedicle and one for each uterine pedicle. No smoke is created and essentially no pneumoperitoneum is lost. The procedure requires only 5-mm ports, which reduces the chances of bowel herniation and potentially results in decreased postoperative pain. Only one port has any exchange of instruments during ligation of the ovarian vessels. Basic suturing techniques are acquired, improving the laparoscopist's skills.

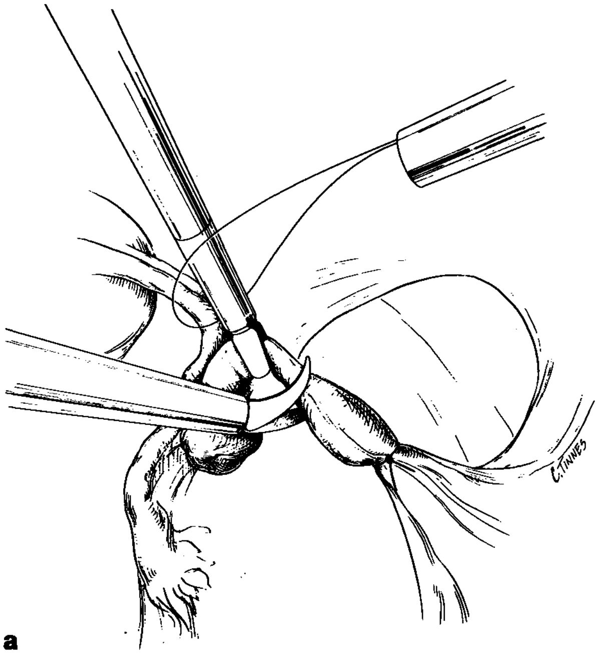

There are some pitfalls with this suturing technique that deserve mention. At times, the proximal end of the infundibulopelvic ligament retracts after transection. This makes it difficult to locate in order to place a second suture. The infundibulopelvic ligament can be cut too close to the first silk suture ligature, allowing this ligature to slide off. The infundibulopelvic ligament can be too wide to be grasped completely with the graspers. Thus, when a

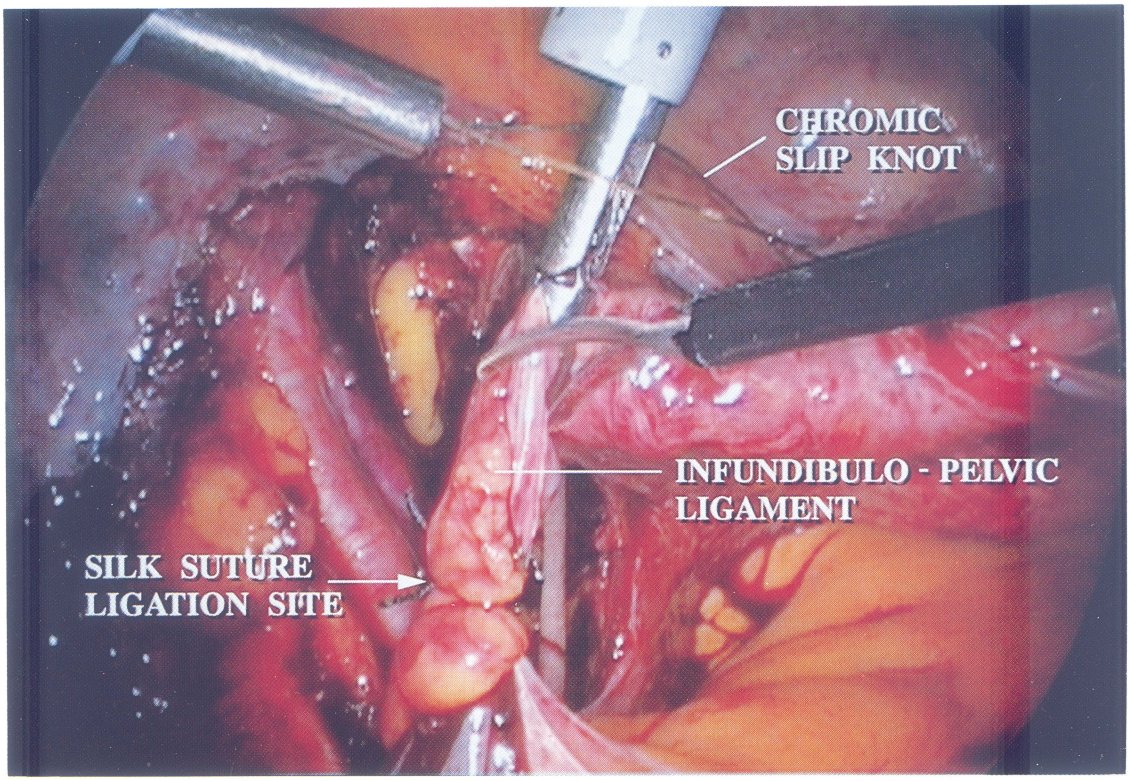

**Figure 4** A. A prefabricated slipknot is placed through the ipsilateral lateral port and a grasper is placed through this slipknot and onto the infundibulopelvic ligament. Scissors are used to cut the ovarian vessels between the grasper and the silk tie. This photograph demonstrates transection of the left ovarian vessels. The grasper is placed through the suprapubic port (top), through the slipknot, and onto the ovarian vessels. The slipknot is placed through the left lateral port. The scissors cut between the silk ligature and the grasper.

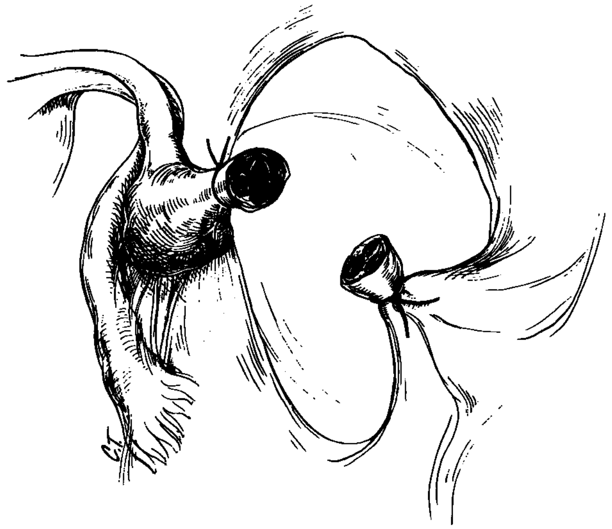

Figure 5 After the ovarian vessels are transected, the prefabricated slipknot is brought around the grasper, and the ovarian vessels are back-tied.

transection is performed between the primary silk ligature and the grasper, some back bleeding can occur until the loop is brought down over the grasper. In addition, the alternation of camera management between the assistant and the surgeon can slow the procedure considerably until the routine is perfected.

Ovarian and uterine artery ligations are both easy to perform quickly when both surgeon and assistant are familiar with the technique. It is more difficult when the surgeon has to talk the assistant through the procedure, particularly uterine artery ligation. If the pedicle is not cut far enough around the grasper tip, one is unable to get the loop tie around the tip of the grasper to secure the pedicle. If the pedicle is cut too far around the grasper tip, it is possible to transect ascending arteries that will not be ligated with the loop tie. Since November 1994 we have ligated uterine arteries in 17 additional patients. Two of these patients had large (14- and 18-week) uteri and both had back-bleeding from the uterine vessels, which required loop ligation. Neither patient required transfusion. Normal-sized uteri, skeletonization of the uterine vessels, and experience minimize bleeding problems with this technique.

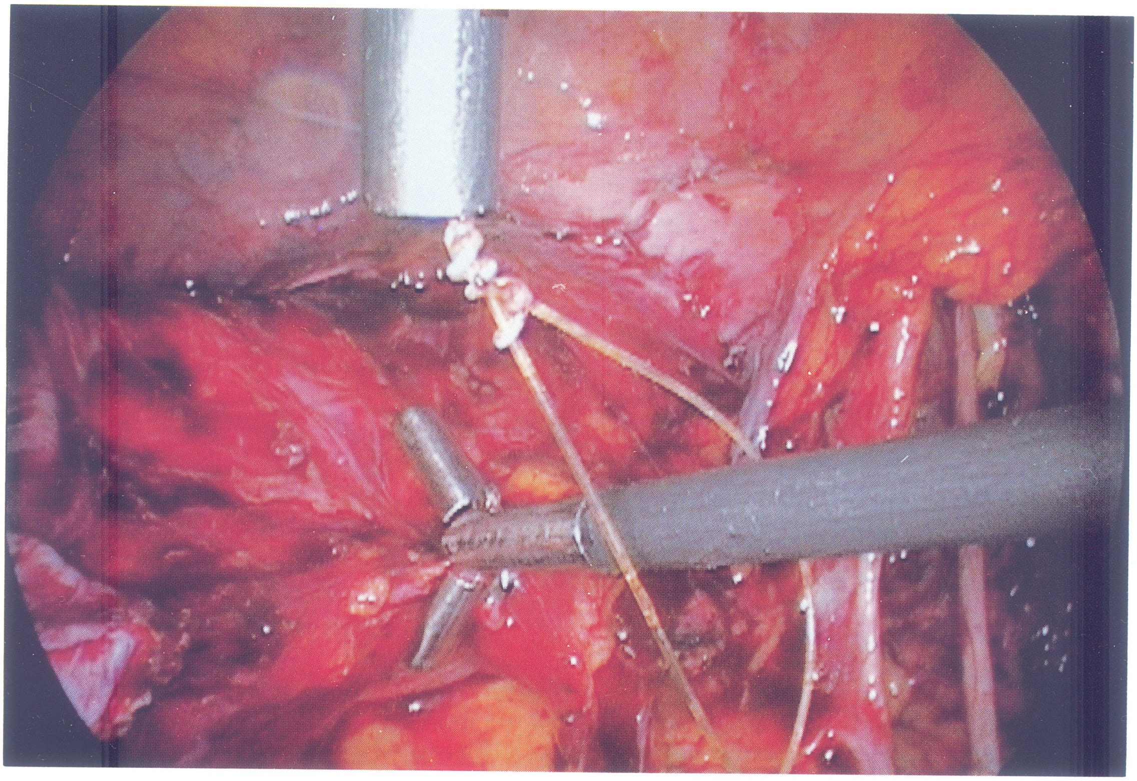

Figure 6 The uterine arteries have been skeletonized on the patient's right side and a grasper coming from the right lateral port is placed through a prefabricated slipknot, which has been placed through the suprapubic port. The grasper grabs the uterine vessels by grasping and sliding off of the cervix.

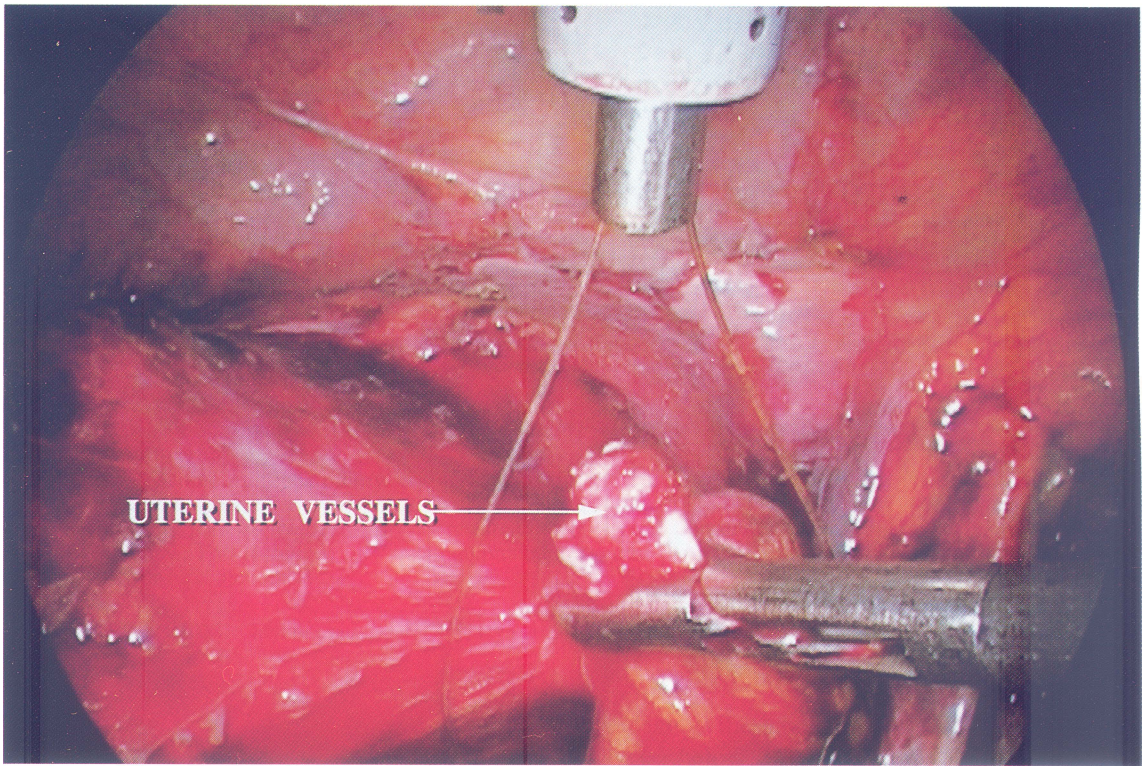

**Figure 7** The uterine vessels have been transected and are ready to be suture ligated with the prefabricated chromic slipknot, which is placed through the suprapubic port.

While the major obstacle to many of today's operative laparoscopic procedures is transection of the vascular pedicles, we believe the major obstacle of tomorrow's procedures will be suturing skills. The simple "grasper-through-the-loop" suture technique we describe will not only broaden the versatility of the endoscopic surgeon, but help to serve as a bridge to accomplishing the advanced suturing techniques, intracorporeal needle and knot-tying, that may be required to manage complications or complete the endoscopic procedures of tomorrow.

### REFERENCES

1. Nezhat F, Nezhat C, Seflen SL. Videolaseroscopy for oophorectomy. Am J Obstet Gynecol 1991;65:1323–1330.
2. Daniell JF, Kurtz BR, Lee JY. Laparoscopic oophorectomy: Comparative study of ligatures, bipolar coagulation, and automatic stapling devices. Obstet Gynecol, 1992;80:325–328.
3. Semm K, Mettler L. Technical progress in pelvic surgery via operative laparoscopy. Am J Obstet Gynecol 1980;138:121–127.
4. Perry CP, Upchurch JC. Pelviscopic adnexectomy. Am J Obstet Gynecol 1990;162:79–81.
5. Langebrekke A, Urnes A. Laparoscopic adnexectomy. Acta Obstet Gynecol Scand 1991;70:605–609.
6. Reich HR. Laparoscopic oophorectomy and salpingo-oophorectomy in the treatment of benign tubo-ovarian disease. Int J Fertil 1987;32:233–236.
7. Montz FJ, Holschneider CH, Munro MG. Incisional hernia following laparoscopy: A survey of the American Association of Gynecologic Laparoscopists. Obstet Gynecol 1994;84:881–884.
8. Boike GM, Miller CE, Spirtos NM, et al. Incisional bowel herniations after operative laparoscopy: A series of 19 cases and review of the literature. Am J Obstet Gynecol 1995;72:1726–1733.
9. Kadar N, Reich H, Lui CY, et al. Incisional hernias after major laparoscopic gynecologic procedures. Am J Obstet Gynecol 1993;168:1493–1495.
10. Woodland MB. Ureteral injury during laparoscopic-assisted vaginal hysterectomy with the endoscopic linear stapler. Am J Obstet Gynecol 1992;167:756–757.
11. Childers JM, Tran AN, Hatch KD, et al. Laparoscopic para-aortic lymphadenectomy in gynecologic malignancies. Obstet Gynecol 1993;82:741–747.
12. Ott DE. Hemoglobin-methemoglobin changes due to laparoscopic smoke production. Presented at the Society of Laparoendoscopic Surgeons' Annual Meeting, Orlando, FL, December 11, 1993.

